# Giant Vulvar Epidermoid Cyst in an Adolescent Girl

**DOI:** 10.1155/2015/942190

**Published:** 2015-04-09

**Authors:** Erbil Karaman, Numan Çim, Zülküf Akdemir, Erkan Elçi, Hüseyin Akdeniz

**Affiliations:** ^1^Department of Obstetric and Gynecology, Yüzüncü Yıl University, 65000 Van, Turkey; ^2^Department of Radiology, Van Training and Research Hospital, 65000 Van, Turkey; ^3^Department of Obstetric and Gynecology, Van Ipekyolu Women and Children Disease Hospital, 65000 Van, Turkey; ^4^Department of Radiology, Van Private Istanbul Hospital, 65000 Van, Turkey

## Abstract

*Introduction*. Vulvar cyst in adolescent girls is very uncommon. Epidermoid cyst can be seen in many sites including face, trunk, and extremities but its occurrence in vulva is uncommon. This is the first case of epidermoid cyst of vulva reported in an adolescent girl.* Case*. A 17-year-old, adolescent girl admitted to our gynecology outpatient clinic with a complaint of painful and palpable mass in her vulva. On examination, a giant mass located in left vulva and labia majora with 11 cm in diameter was seen. The magnetic resonance imaging (MRI) showed a well-defined cystic mass without contrast enhancement. The surgery was advised to the patient and the pathologic examination of mass revealed vulvar epidermoid cyst.* Discussion*. Vulvar cysts generally grow slowly and the main etiologies are vulvar trauma and surgical interventions including episiotomy and female circumcision in some culture. The exact treatment is total surgical excision and pathologic examination. MRI is an important imaging modality for detection of extension to deep perineal tissue and localization of mass in vulva especially in giant ones.* Conclusion*. Although vulvar mass in adolescents is rare, the epidermoid cyst with benign origin should be kept in mind.

## 1. Introduction

The vulvar epidermoid cysts are seen rarely. These cysts usually develop as a result of invagination of superficial squamous epithelium which is an intradermal tumor locating within epidermis [1, 2]. There are various benign cystic masses developing in vulvar site including Bartholin cyst of vulva, epidermoid cyst, Nuck canal cyst, lipoma, and endometrioma [3]. Although, a giant vulvar mass with diameter of up to 15 cm on examination was reported in a recent study [4]. Vulvar epidermal cysts are usually multicystic and slowly growing and a great portion of loculus is less than 1 cm [2]. Historically, vulvar epidermoid cysts are reported to be most commonly involved clitoris which was due to the etiologic factor of female circumcision in some cultures [5, 6]. The main etiologic factors are reported as genital trauma or surgical intervention. The small cysts in vulva without any complaints such as pain or infection require no treatment. The treatment option for a larger or giant vulvar cyst should be total surgical excision which may be difficult and histopathologic examination is required for differentiation from other vulvar lesions.

## 2. Case

A 17-year-old adolescent sexually inactive girl admitted to our gynecology outpatient clinic with complaint of left painful vulvar mass, which had been noticed since the menarche age of 11 years and became painful for last three months. The patient had no history for genital trauma or any surgery. She noticed that the mass was gradually increased in size slowly for a period of approximately 6 years after menarche. Her medical history was unremarkable.

**Figure 1:**
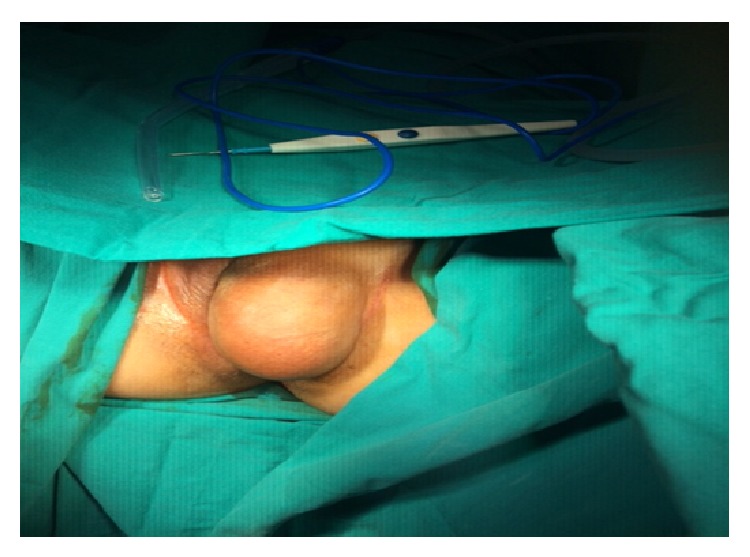
Intraoperative image of giant left vulvar cystic mass.

**Figure 2:**
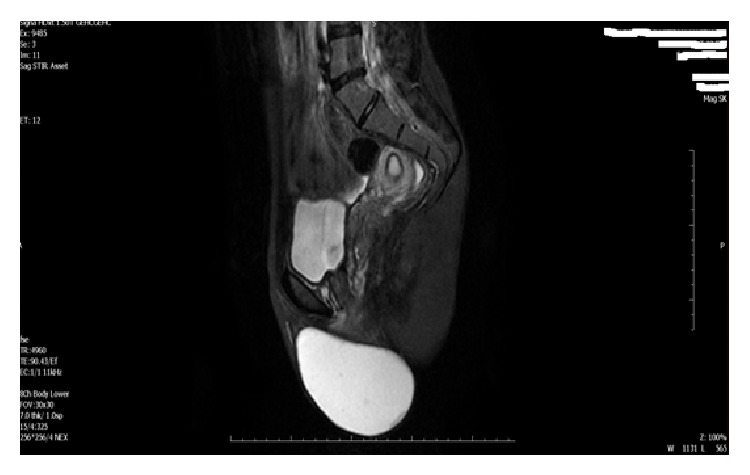
MRI image of 11 × 8 × 10 cm cystic mass with well-defined margins and without contrast enhancement.

Gynecologic examination revealed an 11 cm diameter mobile, regular contoured, and soft but painful mass in left labia majora and vulva. The mass extended to the pubic rami anteriorly, labia majora and clitoris medially, and inner thigh laterally (Figure 1). On suprapelvic ultrasound (USG) examination, uterus, cervix, and adnexal structures were seen normal. USG could not visualize the whole mass. MRI was planned for the differential diagnosis and its extensions into surrounding structures and it revealed an 11 × 8 × 10 cm cystic mass with well-defined margins and without contrast enhancements (Figure 2).

**Figure 3:**
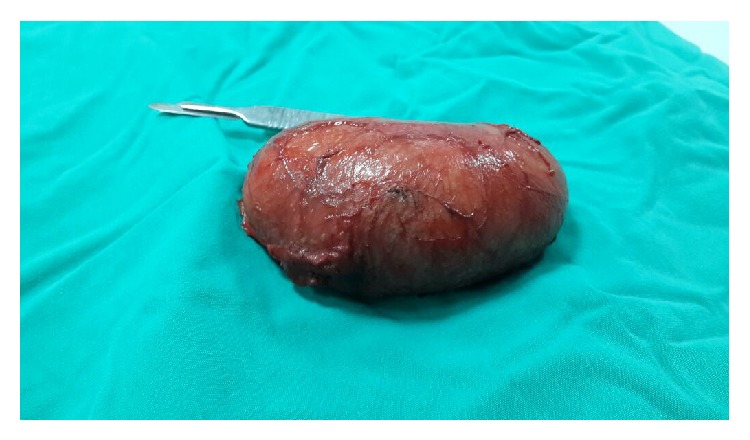
Gross macroscopic pathologic specimen of vulvar cyst.

**Figure 4:**
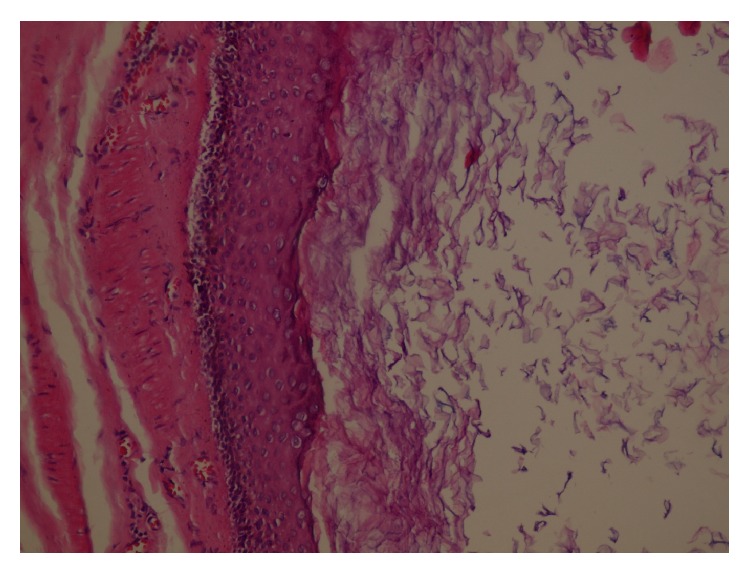
The microscopic image of epidermoid cyst lined with stratified squamous epithelium containing laminated keratinous debris.

The patient underwent surgical operation and the cyst was easily dissected from peripheral tissue without any major bleeding sites and rupture (Figure 3). The histopathologic examination confirmed the diagnosis of epidermoid cyst lined with stratified squamous epithelium containing laminated keratinous debris (Figure 4). The patient was discharged in second postoperative day without any complications. The follow-up examination showed a good cosmetic healing one month after surgery.

## 3. Discussion

Epidermal cyst can arise in many sites including face, trunk, and extremities and also in genital region [7]. Epidermal cyst arises from epidermis displaced into dermis or subcutaneous tissue. Most of the reported data about vulvar epidermoid cyst have been localized in clitoris [8]. The circumcision procedure and trauma have been the main mechanism for clitoral cyst. In our case, there was no history for trauma or genital surgery.

Many cases with reported vulvar epidermal cyst in adolescent girls were located in clitoris [1, 5, 7]. The clitoral cyst causes pain and seeks an early attention for diagnosis but the clinical presentation of vulvar epidermal cyst varies depending on its size and extension to surrounding tissue. It is usually present with slowly growing vulvar mass without any symptoms. It is reported recently by Pehlivan et al. that it can cause difficulty in walking and then could be diagnosed [9]. In our case, the girl was aware of the vulvar mass since menarche which is slowly growing but she did not seek any medical help until the mass became painful. The diameter of the largest epidermal cyst so far reported in the literature has been 12 cm in a 33-year-old woman [4]. Our case was an adolescent girl and the diameter was 11 cm in left vulva including labia majora which is the first case in literature for vulvar epidermoid cyst in an adolescent girl.

Various cystic lesions in vulvar region must be considered in the differential diagnosis. These are Bartholin cyst of vulva, epidermoid cyst, Nuck canal cyst, lipoma, endometrioma, posttraumatic hematoma, and inguinal hernia. Also vulvar malignant tumor should be kept in mind but it is very rare in adolescents [10]. A detailed anamnesis and gynecologic inspection and palpation are very important but not accurate for diagnosis of vulvar epidermal cyst. USG can differentiate a cystic mass and its relationship with clitoris and urinary tract. MRI is a good clinical tool for differentiation of vulvar cystic mass diagnosis, its extension to surrounding tissue, and mass location. A cystic structure is easily depicted by low signal intensities on T1-weighted images and a high signal intensity on T2-weighted images. Contrary to other cysts, epidermoid cysts have the particularity of being hyperintense on diffusion-weighted images [11]. As in our case, the MRI images showed a hyperintense mass on T2-weighted images without any contrast enhancement.

The treatment option for vulvar epidermal cyst is total surgical resection without any major complication. However, if the mass is extending to clitoris or is deep into perineal membrane to inferior hemorrhoidal branches of pudendal vessels, it has the risk of bleeding. The surgeon must be alert of that risk; the hemostatic suture should be in cases of heavy bleeding [4]. Total surgical excision with complete margins was performed in our case without any major bleeding complications. Cosmetic outcome in an adolescent girl after surgery for vulvar mass is an important point especially if she is sexually inactive as was in our case. Careful dissection, closure of wound without tension, and self-shrinkage of vulvar skin during healing are mandatory for good cosmetic outcome [4]. In follow-up, the cosmetic outcome was good as reported by the patient herself one month after surgery.

## 4. Conclusion

As far as we know from literature, up to now, those vulvar epidermal cysts reported in literature were seen in adult and the adolescent cases were involved in clitoral cysts. So this is the largest reported primary vulvar epidermoid cyst arising in an adolescent girl. MRI is a valuable imaging tool for differential diagnosis from other benign cystic lesions. The complete surgical resection is appropriate treatment with good cosmetic results.
